# Combining Technology and Research to Prevent Scald Injuries (the Cool Runnings Intervention): Randomized Controlled Trial

**DOI:** 10.2196/10361

**Published:** 2018-10-10

**Authors:** Jacqueline Burgess, Kerrianne Watt, Roy M Kimble, Cate M Cameron

**Affiliations:** 1 Centre for Children's Burns and Trauma Research University of Queensland Brisbane Australia; 2 Pegg Leditschke Children’s Burns Centre Lady Cilento Childen’s Hospital Brisbane Australia; 3 Wound Management Innovation Cooperative Research Centre Brisbane Australia; 4 College of Public Health, Medical and Veterinary Sciences James Cook University Townsville Australia; 5 Department of Paediatric Surgery, Urology Burns & Trauma Unit Lady Cilento Children's Hospital Brisbane Australia; 6 Jamieson Trauma Institute, Royal Brisbane & Women’s Hospital Metro North Hospital and Health Services District Brisbane Australia; 7 School of Public Health and Social Work Queensland University of Technology Brisbane Australia

**Keywords:** burns, infant, child, mobile apps, gamification, injury, prevention, parent

## Abstract

**Background:**

New technologies, internet accessibility, social media, and increased smartphone ownership provide new opportunities for health researchers to communicate and engage target audiences. An innovative burn prevention intervention was developed using these channels.

**Objective:**

The aim of this study was to evaluate the efficacy of Cool Runnings, an app-based intervention to increase knowledge of childhood burn risk (specifically hot beverage scalds) and correct burn first aid among mothers of young children.

**Methods:**

This was a 2-group, parallel, single-blinded randomized controlled trial (RCT). Participants were women aged 18 years and above, living in Queensland, Australia, with at least 1 child aged 5-12 months at time of enrollment. The primary outcome measures were change in knowledge about risk of burns and correct burn first aid assessed via 2 methods: (1) overall score and (2) categorized as adequate (score=4) versus inadequate (score<4). Efficacy of gamification techniques was also assessed.

**Results:**

In total, 498 participants were recruited via social media and enrolled. At the 6-month follow-up, 244 participants completed the posttest questionnaire. Attrition rates in both groups were similar. Participants who remained in the study did not differ from those lost to follow-up on any characteristics except education level. Although similar at baseline, intervention group participants achieved significantly greater improvement in overall knowledge posttest than control group participants on both primary outcome measures (overall knowledge intervention: mean [SD] of overall knowledge 2.68 [SD 1.00] for intervention vs 2.13 [SD 1.03] for control; 20.7% [25/121] adequate in intervention vs 7.3% [2/123] in control). Consequently, the number needed to treat was 7.46. Logistic regression showed participants exposed to the highest level of disadvantage had 7.3 times higher odds of improved overall knowledge scores than participants in other levels of disadvantage. There were also significant correlations between gamification techniques and knowledge change (*P*<.001). In addition, odds of knowledge improvement between baseline and 6-month follow-up was higher in participants with low-moderate app activity compared with no app activity (odds ratio [OR] 8.59, 95% CI 2.9-25.02) and much higher in participants with high app activity (OR 18.26, 95% CI 7.1-46.8).

**Conclusions:**

Despite substantial loss to follow-up, this RCT demonstrates the Cool Runnings app was an effective intervention for improving knowledge about risks of hot beverage scalds and burn first aid in mothers of young children. The benefits of combining gamification elements in the intervention were also highlighted. Given the low cost and large reach of smartphone apps to deliver content to and engage with targeted populations, the results from this RCT provide important information on how smartphone apps can be used for widespread injury prevention campaigns and public health campaigns generally.

**Trial Registration:**

Australian New Zealand Clinical Trials Registry ACTRN12616000019404; https://www.anzctr.org.au/Trial/Registration/TrialReview.aspx?id=369745&showOriginal=true&isReview=true (Archived by WebCite at http://www.webcitation.org/72b1E8gTW)

## Introduction

### App Technology

Advances in technology, expansion of internet access, and increased mobile phone ownership globally have led to a new channel for disseminating health information and engaging with large or specific populations. With the popularity of smartphones, there has been a proliferation of smartphone apps—6 million in the 2 leading app stores (Google Play: 2.8 million, Apple app store: 2.2 million) [1]. Of these, 259,000 are health-related apps [2]. Increasingly, apps are being used by health agencies and researchers to gather and present information to study participants and the general public. There is a growing body of evidence showing the successful use of smartphone apps to encourage healthy habits such as increasing physical activity [3] and promoting weight loss [4], managing chronic diseases [5,6], and delivering mental health programs [7]. One area that has not yet been studied is the use of this technology in injury prevention.

### Childhood Burns

Childhood burns are serious injuries that can result in substantial pain and suffering and lead to life-long scarring and surgical procedures as the child grows. The physical, emotional, and financial burden to the child and family can be significant [8,9]. The leading cause of childhood burns in developed countries is hot drink scalds [10-13]. In Australia, hot drink scalds account for 18% of all childhood burns [14,15]. This injury peaks in children aged 6 to 18 months, usually occurs in the child’s home, and is witnessed by the parent or supervising adult [13-17]. Given these facts, an app-based prevention intervention was developed to target mothers with children aged 5 to 12 months about risks of hot drink scalds, as well as the correct first-aid treatment to apply, should a burn occur.

### Cool Runnings

On the basis of the Health Belief Model [18], the aim of the Cool Runnings randomized controlled trial (RCT) was to assess the impact of a contemporary app-based public health campaign using gamification on knowledge about child burns. Specifically, the aim of the intervention was to increase knowledge of the primary carer about the severity and frequency of hot drink scalds, provide them with developmental-stage messages on how to protect their child and intervene, and finally the correct burn first-aid treatment to apply should a burn occur. The 2 aims of this study were therefore to (1) assess change in knowledge from baseline to follow-up in the intervention group compared with the control group and (2) investigate the impact of level of app engagement on change in knowledge from baseline to follow-up.

## Methods

### Study Design

This study was a 2-group, parallel, single-blinded RCT of an app-based prevention and first-aid education intervention for burns. This study was registered with the Australian and New Zealand Clinical Trials Registry (ACTRN12616000019404). The full study protocol has been published previously [19].

### Study Setting

Participants from Queensland, Australia, were recruited. Eligibility criteria were females aged 18 years and above; who resided in Queensland, Australia; and had at least 1 child aged 5 to 12 months at enrollment. Ownership of a smartphone was required for intervention delivery. Study duration was 6 months.

### Recruitment

Participants were recruited via online social media advertisements, specifically through Facebook and Instagram, between January 2016 and February 2016. A detailed description of the recruitment process for this study has been published previously [20].

### Randomization

Computerized sequence generation was used to randomize participants. Randomization was stratified by maternal age (18-28 years and 29+ years) based on the mean national maternal age [21].

### Blinding

Participants were blinded to their allocation group (the terms *blue group* and *green group* were used). Study investigators assessed the outcome data collected in pre- and postquestionnaires in a blinded format. However, blinding was not possible for analyzing the results of gamification techniques, as they only applied to the intervention group.

### Intervention

Participants in the intervention group were compared with those in the control group. Both groups accessed an app throughout the study, but in the intervention group, gamification techniques were incorporated into the app. The control group accessed a slightly different version of the app with no gamification.

#### Intervention Group

**Figure 1:**
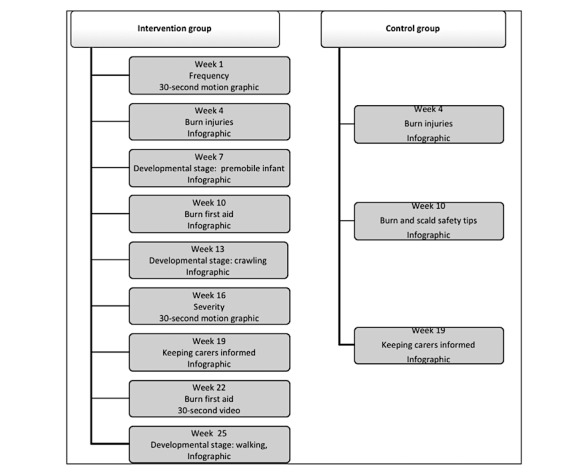
Content calendar for the Cool Runnings intervention (source: Burgess et al [19]).

During the 6-month intervention, participants allocated to the intervention group received 9 intervention messages via the app related to risks of hot beverage scalds, risks of developmental stage–based burns, and burn first-aid treatment (illustrated in Figure 1). These messages were provided in a variety of mediums (infographics, 30-second videos, and motion graphics) at 3-week intervals. In between these messages, participants were given opportunities to engage with the app through activities such as answering pop quizzes and completing missions (such as photo uploads) that reinforced each of the intervention message themes. Gamification techniques were used to keep participants engaged and active on the app. Each time participants viewed a message, correctly answered a quiz question, or uploaded a photo, they were rewarded with points. Accrued points were displayed on weekly leaderboards in the app, and once a certain number of points were reached, they could be redeemed for rewards, such as shopping and movie vouchers.

#### Control Group

This group accessed a slightly different app interface; no gamification techniques were used with this group. Participants from the control group only received 3 messages during the 6-month intervention. These messages were infographics, and there were no opportunities for participants to engage with the material.

### Data Collection

Baseline and 6-month follow-up questionnaires were completed by participants in the intervention and control groups. The baseline questionnaire included demographic factors (such as education level, age of youngest child, number of children, marital status, and smoking status). Place of residence postcodes were also collected and later recoded using the Accessibility/Remoteness Index of Australia (ARIA) 2011 data [22], and the Socio-Economic Indexes for Areas (SEIFA) [23] as measures to broadly assess socioeconomic status (SES). The SEIFA data were based on aggregate area-level SES disadvantage indicators and were categorized into quintiles (1=most disadvantaged and 5=least disadvantaged). The ARIA is a measure of geographical remoteness, categorized as urban, periurban, and remote.

The questionnaires also included the extent of hot beverage scald risk awareness (2 questions) and burn first aid knowledge (2 questions). Full baseline data from this study are described elsewhere [24].

The 6-month follow-up questionnaire repeated the questions relating to risks of hot beverage scalds and burn first aid knowledge. Participant engagement with the app, including number of app opens, content views, and gamification activities by participants in the intervention group were recorded by the app.

### Primary Outcome Measures

The primary outcome for this study was change in knowledge based on a 4-point knowledge score measured by 3 components:

1. Knowledge of correct burn first aid,
2. Knowledge of the main cause of burns or scalds in children aged 0 to 15 years, and
3. Knowledge of the main age group at risk of these burns or scalds.

Two questions were used to determine burn first aid knowledge: an open-ended question (“What is the recommended first aid treatment for a burn or scald?”) and a multiple-choice question regarding duration for applying cool running water. Correct first aid knowledge was defined as cool running water for 20 min based on clinical evidence of benefit [25]. Participants who responded with “20-minutes cool running water” to the open-ended question were allocated 2 points. Participants whose answer involved cool running water but mentioned an incorrect or no time and who then responded to the multiple-choice question on duration correctly were awarded 1.5 points; only 1 point was awarded if the subsequent multiple-choice question on duration was incorrect. All other responses (such as flour and ice), including “don’t know,” were allocated 0 points.

Knowledge of main cause of burns or scalds in children aged 0 to 15 years was assessed via a multiple-choice question. Anything other than hot drink scalds (1 point) was coded incorrect (0 points). Knowledge of main age group at risk of these burns or scalds was also assessed via multiple-choice questions. Anything other than 0 to 2 years (1 point) was coded incorrect (0 points).

One final overall knowledge score was then computed, which combined the responses to the main cause of burns, age group most at risk, and burn first aid knowledge, to yield a total possible score of 4. The change in total score on *overall knowledge* between baseline and postintervention was calculated.

The score was also recoded to a binary variable. Participants who received a score of 4 were coded as *adequate knowledge,* and all other participants were coded as *inadequate knowledge*. Participants whose responses moved from *inadequate* to *adequate* at 6-month follow-up were categorized as *improved*. All other participants were coded as *no improvement*.

In addition, burn first aid knowledge was categorized into a binary variable: adequate (cool running water for 20 min in the open-ended question) versus inadequate (any other response). Participants whose responses moved from *inadequate* to *adequate* at 6-month follow-up were categorized as *improved*. All other participants were coded as *no improvement*.

### Gamification and App Engagement

The app accessed by the intervention group incorporated gamification techniques. Participants in the intervention group were encouraged to earn points by viewing content, completing pop quizzes, and uploading photos as part of weekly challenges. Weekly leaderboard and challenge winners were awarded additional bonus points. Level of engagement was measured across following 4 dimensions: the frequency of opening the app (app views: intervention and control groups), frequency of content views (intervention and control), number of pop quiz completions (intervention), and participation in photo-sharing activities (intervention). These numerical and continuous variables were each then categorized into none, low-moderate, and high. High engagement occurred if the participant engaged with at least 2 out of 3 of the available engagement opportunities for that element (eg, there were 6 opportunities to upload photos, so participants were required to upload 4 photos to be coded as high engagement).

One final measure of app engagement was then derived for all participants (intervention and control) as follows: no engagement on any of the 4 elements other than opening the app; high engagement (viewing content 4 or more times, sharing photos 4 or more times, or completing quizzes 9 or more times); and low-moderate engagement (any other level of engagement).

### Data Analysis

All statistical analyses were conducted using SPSS version 24 (IBM Corporation, Armonk, NY, USA). Descriptive analyses were completed to determine whether there were any between-group differences (intervention vs control) at baseline on demographic characteristics and the primary outcome measure. Chi-square tests were used for categorical variables, and independent sample *t* tests were used for numerical variables [26]. Specifically, an independent sample *t* test was used to assess between-group differences on change in overall knowledge score at 6-month follow-up as a function of the intervention. A chi-square test was performed to determine whether the proportion of participants with improved knowledge differed between intervention and control groups. Event rate of improved overall knowledge (all 4 responses correct) was also calculated for the intervention and control groups. Subsequently, the number needed to treat (NNT) was calculated. Correlations were performed to determine whether the 4 separate elements of app engagement were related to each other. Alpha of .05 was used in the interpretation of all descriptive analyses. Univariate logistic regression analyses were conducted to determine whether there were any significant independent predictors of knowledge improvement (no improvement vs improvement). Potential predictor variables were intervention status (intervention vs control) and demographic variables (education level, age of youngest child, age of respondent, number of children, marital status, smoking status, ARIA category, SES as measured through SEIFA, and first-time mother).

Any variables where *P*<.20 was obtained in univariate logistic regression analyses were then entered into 1 adjusted model. If a variable was not significantly associated with the outcome in the multivariate model, it was removed and the impact on all remaining variables was assessed. If the odds ratio (OR) for any other variables in the model changed more than 10%, the variable was retained in the model as a potential confounder. If not, it was removed. This process was repeated until there were no variables with *P*>.05 in the model or removing the nonsignificant variables from the model did not create changes of greater than 10% to the ORs of variables remaining in the model.

To investigate the impact of level of app engagement on change in knowledge from baseline to follow-up, univariate analyses were first completed using the 4 numerical measures of engagement for the intervention group only (frequency of app views, frequency of content views, number of pop quiz completions, and number of times participated in photo-sharing activities). Afterward, for all participants (intervention and control), univariate analyses were completed on the final composite measure (no engagement, moderate engagement, and high engagement). Subsequently, an additional multivariate analysis was completed using this composite measure of app engagement as one of the predictor variables, instead of intervention status—the same demographic variables described above were used—and the same process followed. Analyses completed on any follow-up data were conducted on a *per protocol* basis.

## Results

**Figure 2:**
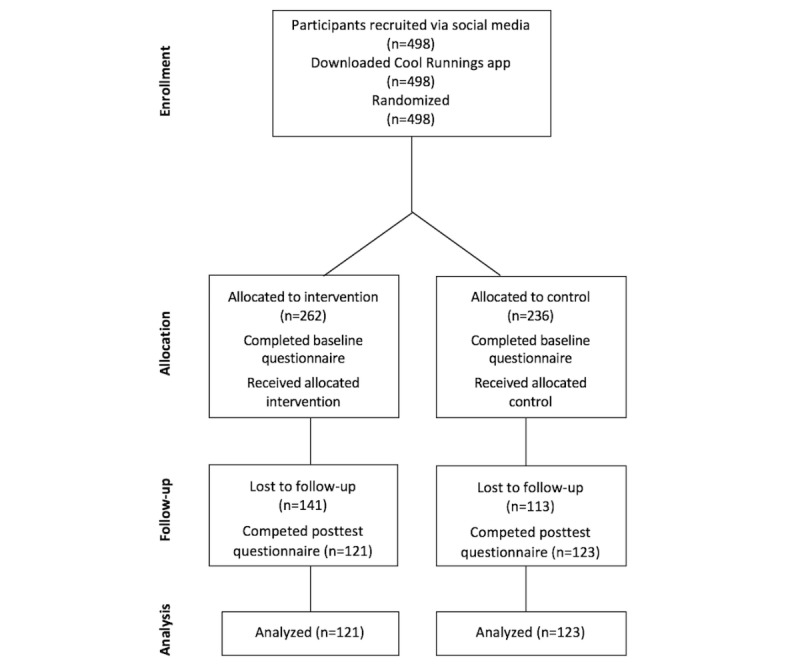
Flowchart of participants through each stage of the randomized controlled trial.

Figure 2 illustrates the flow of participants through the study. A total of 498 participants were enrolled in the Cool Runnings study: 262 in the intervention group and 236 in the control group. After the 6-month intervention, 121 intervention participants (121/262, 46.1%) and 123 control participants (123/236, 52.1%) completed the posttest questionnaire.

### Trial Retention

The trial experienced 51% attrition overall. Attrition rates in both groups were similar: (intervention: 141/262, 53.8%; control: 113/236, 47.8%). Participants who remained in the study did not differ from those who were lost to follow-up on any baseline characteristics except for education level. A higher proportion of participants who remained in the study had a university degree (28.7%; n=70) than those who were lost to follow-up (16.5%; n=42; χ^2^_4_=15.8; *P*=.003). Mean overall knowledge was higher at baseline in participants (mean 2.06 [SD 0.87]) than in those who were lost to follow-up (mean 1.93 [SD 0.87]), but this difference was not significant (*t*_490_=1.72; *P*=.09).

Within-group analyses were also conducted to see whether there were differences in participants who completed the study and those who were lost to follow-up. There was no difference between participants who completed the study and those who were lost to follow-up in relation to proportion with adequate overall knowledge (score of 4) versus inadequate (score<4) in the intervention group (*P*=.62) or in the control group (*P*=.99). However, among the participants allocated to the intervention group, overall knowledge at baseline was significantly higher in those who remained in the study (mean 2.12 [SD 0.84]) than in those who did not complete the study (mean 1.84 [SD 0.87]; *t*_258_=2.64; *P*=.009). The remainder of the analyses were completed by *compliance only*.

### Primary Outcome Measures

At baseline, there were no differences in any demographic or other sample characteristics between the intervention and control groups (*P*>.05; see Table 1 for actual *P* values). Importantly, there were no differences in the mean total knowledge score (*P*=.54), the proportion of participants who demonstrated adequate overall knowledge (*P=*.49), or in any of the dimensions comprising the score (burn risk knowledge [*P=*.93] and burn first aid knowledge [*P=*.57]; Table 2).

**Figure 3:**
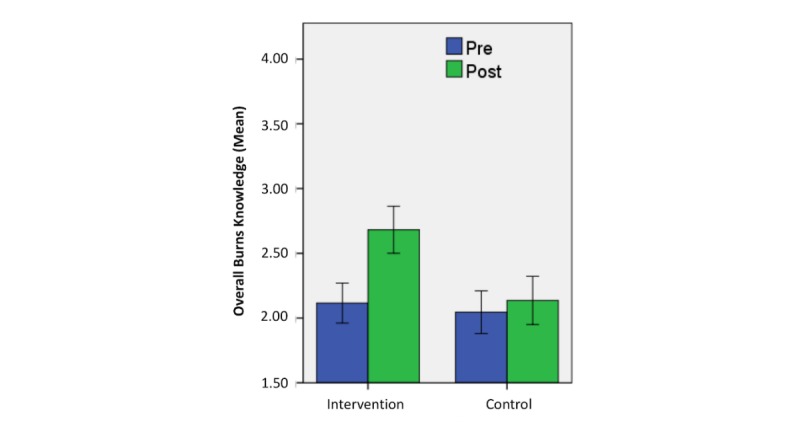
Change in mean overall knowledge score between baseline and 6-month follow-up in intervention and control groups (error bars: 95% CI).

Changes in overall knowledge of participants between baseline and 6-month follow-up are shown in Table 2 and Figure 3. Although similar at baseline, intervention group participants achieved significantly greater improvement in overall knowledge posttest than control group participants (*t*_240_=3.37; *P*<.001; Figure 3). *Event rate* of improved overall knowledge (change from inadequate at baseline to adequate at 6-month follow-up) was significantly higher in the intervention group (25/121, 20.7%) than in the control group (9/123, 7.3%) (χ^2^_1_=9.1; *P*=.003). Consequently, the NNT was 7.46. That is, 8 people needed to be exposed to this intervention to improve inadequate overall knowledge to adequate knowledge (ie, score of <4 to a score of 4) in 1 additional person. A sensitivity analysis was completed with respect to the event rate and NNT. First, the event rate was recalculated assuming that all participants who were lost to follow-up did not improve their score (ie, demonstrated inadequate knowledge at baseline and at follow-up). The event rate of improved overall knowledge was 9.5% in the intervention group and 3.8% in the control group. The NNT was 17.5. Next, the event rate was recalculated assuming that all participants who were lost to follow-up did improve their score from inadequate at baseline to adequate at follow-up (intervention: 63.35%; control: 51.69%). The NNT was 8.57.

### Demographic Predictors of Overall Knowledge of Burns

Univariate logistic regressions indicated that the following variables were related to improvement in overall knowledge (from inadequate to adequate) between baseline and follow-up: being in the intervention group, age of respondent, SES as measured through SEIFA quintile, and remoteness (as measured by ARIA category). These variables were entered into 1 multivariate model ,and nonsignificant variables were removed one at a time, assessing the impact on remaining variables. In the final model, the only variables that were significantly associated with the improvement in overall knowledge were being allocated to the intervention (OR 3.3, 95% CI 1.4-7.7) and SES as measured through SEIFA. Specifically, odds of improving overall knowledge scores were higher in participants whose postcode indicated they were exposed to the highest level of disadvantage (OR 7.30, 95% CI 1.2-42.9) compared with participants exposed to the lowest levels of disadvantage (ie, highest advantage). Age and remoteness (as measured by ARIA category) were not significantly associated with improved knowledge; however, they were retained in the model because there was evidence of confounding (ie, removing these variables from the model changed the ORs of other variables in the model more than 10%).

### Gamification and App Engagement

Gamification and app use activity (app views, content views, pop quiz completions, and photo sharing) were also measured. Participants in the intervention group earned points each time they viewed content, correctly answered pop quiz questions, and uploaded photos. Winners of the weekly photo mission won additional bonus points. In total, 58 participants in the intervention group accrued sufficient points to redeem their points for movie or supermarket vouchers; however, only 3 participants took advantage of this. The leaderboard (only available to the intervention group) showed that participants’ points were viewed 535 times.

The mean number of app opens for the intervention group was 18.31 (SD 42.1; minimum 1; maximum 347; median 5.0; interquartile range [IQR] 13.5) and 5.03 (SD 5.28; minimum 1; maximum 28) for the control group (median 3.0; IQR 4.0). Overall, 1 participant from the intervention group opened the app a total of 347 times; however, the majority of participants opened the app 10 times or less in both the intervention group (69%) and control group (65%). Mean content views for the intervention group was 1.96 (SD 2.86; median 0.0, IQR 4.5) and 0.98 (SD 0.77) for the control group (median 0; IQR 0). The mean quiz completions for the intervention group was 2.45 (SD 4.33), the median was 0 (IQR 2.5), and the mean number of photos shared was 2.23 (SD 5.11; median 0; IQR 2.0). With respect to the composite measure of app engagement, 27.3% (33/121) of the intervention group and 1.6% (2/123) of control participants were categorized as high engagement and 51.2% (62/121) of the intervention group had no app engagement (62) versus 98.4% (121/123) of the control group.

Univariate logistic regression analyses (intervention group only) showed that each of the 4 (numerical) measures of engagement were significantly associated with improvement in overall knowledge from inadequate at baseline to adequate at 6-month follow-up (quiz total: OR 1.34, 95% CI 1.2-1.5; content: OR 1.49, 95% CI 1.3-1.7; app opens: OR 1.05, 95% CI 1.02-1.07; and photo uploads: OR 1.33, 95% CI 1.1-1.6). The 4 elements of app engagement were strongly correlated with each other (see Table 3).

**Table 1:** Demographic characteristics and knowledge of risks of scalds and first aid in intervention and control groups at baseline.

Characteristics		Intervention (n=121), n (%)	Control (n=123), n (%)	*P* value
**Age of participant (in years)**				.56
	18-24	19 (15.7)	20 (16.3)	
	25-29	34 (28.1)	43 (35.0)	
	30-34	46 (38.0)	44 (35.8)	
	35+	22 (18.2)	16 (13.0)	
**Marital status**				.21
	Single	15 (12.4)	9 (7.3)	
	Married	76 (62.8)	70 (56.9)	
	De facto	28 (23.1)	40 (32.5)	
	Separated or divorced	2 (1.7)	4 (3.3)	
**Highest education level**				.62
	Less than year 12	13 (10.7)	22 (17.9)	
	Year 12 completion	27 (22.3)	25 (20.3)	
	Technical and further education certificate or advanced diploma	34 (28.1)	33 (26.8)	
	University degree	37 (30.6)	33 (26.8)	
	Postgraduate degree	10 (8.3)	10 (8.1)	
**Current smoker**				.70
	Smoker	23 (19.0)	21 (17.1)	
	Nonsmoker	98 (80.9)	102 (82.9)	
**Country of birth**				.41
	Australia	104 (85.9)	101 (82.1)	
	Other	17 (14.0)	22 (17.9)	
**SEIFA^a^**				.92
	1 (most disadvantaged)	10 (8.3)	10 (8.2)	
	2	20 (16.5)	16 (13.1)	
	3	32 (26.4)	30 (24.6)	
	4	46 (38)	51 (41.8)	
	5 (least disadvantaged)	13 (10.7)	15 (12.3)	
**ARIA^b^**				.64
	Urban (major cities)	62 (51.2)	62 (50.8)	
	Periurban (inner or outer regional)	44 (36.4)	49 (40.2)	
	Remote or very remote	15 (12.4)	11 (9.0)	
**First-time mothers**				.50
	Yes	48 (39.7)	54 (43.9)	
	No	73 (60.3)	69 (56.1)	
**Number of children in the household**				.25
	1 child	53 (43.8)	63 (51.2)	
	More than 1 child	68 (56.2)	60 (48.8)	

^a^SEIFA: Socioeconomic Index for Areas. SEIFA was used to estimate socioeconomic status in this study. Specifically, the Index of Relative Socioeconomic Advantage and Disadvantage. Higher deciles reflect higher relative advantage, and lower deciles reflect lower relative advantage. Deciles were reduced to 5 categories.

^b^ARIA: Accessibility/Remoteness Index of Australia. Location of usual residence was categorized using ARIA, developed by National Centre for the Social Applications of Geographic Information Systems. Each geographical area was allocated a score between 0 and 15, based on the (road) distance to nearby towns that provide services. Scores were then allocated to the following categories (Office of Economic and Statistical Research Queensland, 2011): urban (major city: 0.0-0.2); periurban (inner regional: 0.2-2.4 and outer regional: 2.4-5.92); and remote (remote: 5.92-10.53; very remote: 10.53+).

**Table 2:** Change in overall knowledge and burn first aid at 6-month follow-up.

Knowledge metrics		Intervention (n=121)		Control (n=123)	
		Pretest^a^	Posttest	Pretest	Posttest
Overall knowledge, mean (SD)		2.11 (860)	2.68 (1.00)	2.04 (0.915)	2.13 (1.03)
Overall knowledge^b^—adequate, n (%)		3 (2.5)	27 (22.3)	5 (4.1)	12 (9.8)
Main cause of burns or scalds—correct, n (%)		40 (33.1)	72 (59.5)	44 (35.8)	57 (46.3)
Age group most at risk of burns or scalds—correct, n (%)		64 (52.9)	72 (59.5)	56 (45.5)	48 (39.0)
**Burn first aid knowledge, n (%)**					
	Adequate (cool running water for 20 min, unprompted)	12 (9.9)	48 (39.7)	15 (12.4)	32 (26.0)
	Inadequate (all other responses)	109 (90.1)	73 (60.3)	108 (87.6)	91 (73.9)

^a^Participants lost to follow-up are not included in the Pretest column.

^b^Proportion of participants who had *adequate* overall knowledge at baseline and 6-month follow-up are shown in the table. These rates are slightly different from the event rates that were calculated for improvement in overall knowledge between baseline and follow-up (25 [20.7%] participants in the intervention group improved overall knowledge between baseline and follow-up compared with 9 [7.3%] people in the control group). Only those participants who demonstrated improvement from inadequate knowledge at baseline to adequate knowledge at 6-month follow-up were included in the *improved knowledge* group).

**Table 3:** Intercorrelations between measures of app engagement (N=244).

App activities	Change in knowledge	Quiz completion	Content view	App opens
Quiz	.48^a^	—	—	—
Content	.44^a^	.86^a^	—	—
App	.35^a^	.72^a^	.56^a^	—
Photo uploads	.40^a^	.83^a^	.65^a^	.76^a^

^a^*P*<.001.

Univariate logistic regression analyses (intervention and control groups) indicated that the composite measure of app engagement was associated with change in knowledge at 6-month follow-up. Odds of improved overall knowledge from inadequate to adequate were significantly higher in participants who demonstrated low-moderate app engagement (OR 8.59; 95% CI 2.9-25.02) and high app engagement (OR 18.26; 95% CI 7.1-46.8) than participants with no engagement. The composite measure of app engagement was entered into a multivariate logistic regression model with the variables previously identified in univariate analyses as significantly associated with the primary outcome measure (age of respondent, SES as measured through SEIFA quintile, and remoteness as measured by ARIA category). Nonsignificant variables were removed from the model one at a time and the impact on remaining variables was assessed. In the final model, the only variable that was significantly associated with improvement in overall knowledge was app engagement (low-moderate: OR 6.81; 95% CI 2.2-21.4 and high: OR 33.84; 95% CI 10.6-107.6). Age of respondent, remoteness (as measured by ARIA category), and SES (as measured through SEIFA) were not significantly associated with app engagement; however, they were retained in the model because there was evidence of confounding (ie, removing these variables from the model changed the ORs of other variables in the model more than 10%).

## Discussion

### Principal Findings

This RCT has demonstrated the Cool Runnings app to be an effective intervention for improving knowledge about risks of hot beverage scalds and burn first aid in mothers of young children. Only 8 people needed to be exposed to this intervention to improve inadequate overall knowledge to adequate knowledge in 1 additional person. Hot beverage scalds present a major pediatric public health issue that requires attention and prevention efforts, and this RCT details the implementation and evaluation of innovative methods and techniques to address this injury.

To our knowledge, this is the first study to evaluate an app-based delivery of injury prevention messages and the first study to gauge the efficacy of gamification in an injury prevention intervention. Given the low cost and large reach of smartphone apps to deliver content to and engage with targeted populations, results from this RCT provide important information on how smartphone apps can be used for widespread injury prevention campaigns. This study looked specifically at the use of this technology in a prevention campaign aimed at hot beverage scalds, the leading cause of childhood burn injuries. Although numerous studies have reported the high incidence of this injury, there is a paucity of interventions aimed at preventing them.

There have been few success stories when it comes to prevention campaigns for childhood burns [27], apart from those that have included passive approaches that have the benefit of legislative, engineering, and design support, such as hot water tempering valves and flame-retardant children’s sleepwear [28-30]. Certainly, prevention campaigns for burns that focus solely on education have had little demonstrated success. Due to this and because education is the most likely strategy to be effective for hot beverage scald prevention (because of limited capacity for other approaches such as engineering or environmental approaches to work), a novel approach was followed to develop and implement the Cool Runnings intervention.

In addition to being allocated to the intervention group, SES (as measured by SEIFA through postcode of residence) was significantly inversely associated with improvement in knowledge score relative to baseline. This is encouraging, given the recognized disparities in burn incidence and first aid knowledge and use among those who are socioeconomically disadvantaged [31-34]. Some burn prevention campaigns have targeted these specific groups with mixed results [35,36]. The growing global ownership of smartphones and the promise of app-based technology may change this. In 2016, the average global ownership of smartphones was 81% (77% of US adults and 84% Australian adults) [37]. Lack of other significant predictors of increased burn knowledge may be interpreted as an indication of the success of the intervention across the target group. There was a broad sample of participants included in this study—older and younger primigravid mothers, with various levels of education, and from regional, rural, and remote locations. Participants were representative of the target population (women who delivered in Queensland in 2015) with regard to age, marital status, being a first-time mother, and country of birth [38].

Smartphone ownership goes beyond socioeconomic, racial, and ethnic boundaries, with a report by the Pew Institute [39] showing that more than half of most sociodemographic groups own a smartphone. Smartphones provide the opportunity to engage with people wherever they are and whenever it suits them to see a message. The use of smartphones to deliver information in a way that is interactive and engaging, rather than a one-way flow of static communication, is also compelling. App-based campaigns can cater to participants’ different learning styles—whether it’s visual, auditory, or kinesthetic—through the delivery of various message types, such as animations, videos, or infographics, and make it more appealing to a diverse audience.

This study also showed an association between change in knowledge and the gamification strategies used. Gamification takes the gaming principles of rewards, competition, and personalization to engage participants and motivate them toward preferred behaviors. Gamification is widely used in business to increase loyalty and create long-term engagement, but the evidence of its efficacy in changing health behaviors is still in its infancy. Gamification is commonly used in workplace health initiatives [40], and the trend of using gamification techniques and strategies in health-related apps is burgeoning. However, theoretical frameworks are still being developed, and there remains relatively little scientific literature as to its efficacy in improving health behavior outcomes [40,41]. The aim of incorporating gamification elements to health-related nongames is to improve user experience and engagement while increasing intrinsic motivators likely to result in the adoption of a behavior or knowledge change [42,43]. Extrinsic and intrinsic rewards were used in this intervention, with the potential to win financial rewards and prizes (extrinsic), and learn how to protect their child from injuries and/or keep children safe (intrinsic). In this study, participants in the intervention group were *gamed* into viewing content, uploading photos, and completing pop quizzes with the ability to earn points for each activity. These points accumulated and were displayed on the weekly leaderboard on the app. Although 58 participants accrued enough points to redeem them for tangible rewards (Aus $25 and Aus $50 movie or shopping vouchers), only 3 participants did so. Reasons for this may be the intangible rewards (such as leaderboard position and weekly photo winner badges) were of more perceived value, or the motivation may have been more competition-based rather than incentive-based. This finding shows potential for larger widespread campaigns in which providing ongoing tangible financial rewards would make scalability difficult. The results of this study suggest the inclusion of gamification strategies in injury prevention and public health campaigns could lead to improved results. Unfortunately, the use of gamification techniques did not appear to help with retention of participants. One reason for this may be the length of the intervention. It is difficult to know at what specific point during the intervention period participants dropped out. The lack of personal contact associated with an app-based intervention may also have contributed to the attrition rate. Future studies should test shorter intervention periods to determine optimal duration.

The loss to follow-up in this RCT provides some important information about the challenges associated with recruiting and retaining participants using this technology. Overall, the potential differences in the participants versus nonparticipants with respect to burn first aid knowledge and the differential loss to follow-up in the intervention group of those with lower knowledge indicates that further research is required regarding those with lower knowledge (who would most benefit from an intervention to improve knowledge), and how to engage or retain their interest and participation. Sensitivity analyses were conducted to further understand the potential impact of the loss to follow-up. When it was assumed that all participants who were lost to follow-up improved their knowledge score from inadequate to adequate, the NNT was 8.57. When it was assumed that all participants lost to follow-up did not improve their knowledge score, the NNT was 17.5 (compared with NNT of 7.46 calculated for the participants who were retained in the study).

### Limitations

This study has several limitations. In both the control and intervention groups, there was a large loss to follow-up (48.9%). This loss to follow-up raises the potential for attrition bias. However, the attrition rate in both groups was similar (54% vs 48%), and participants did not differ significantly from those who were lost to follow-up on most of the measured characteristics. The exception was education (participants who remained in the study demonstrated a higher level of education). In addition, participants originally allocated to the intervention group who completed the study demonstrated a significantly higher baseline overall knowledge score than those who were lost to follow-up. This did not occur in the control group. Interestingly, there was no difference in the proportion of participants versus dropouts who demonstrated adequate versus inadequate knowledge. It is also acknowledged that there may have been differences between participants who remained in the study and those who were lost to follow-up that were not measured in the survey. Given the novelty of this intervention, and in particular within this context, we intentionally conducted a *per protocol* analyses to demonstrate efficacy of the app, and this may be considered a limitation of the analyses, although sensitivity analyses were conducted to further understand the potential impact of the loss to follow-up. The relatively small numbers involved in this study mean that the multivariate analyses on demographic variables associated with change in knowledge (especially when app engagement is considered) and the analyses on predictors of app engagement should be interpreted with caution.

Postcodes were used as a proxy for SES in this study. Hence, this measure may not have been representative of the individual-level SES of participants. It is possible that other factors external to study participation (such as enrollment in a first aid course) may have contributed to the observed change in outcome. However, this was an RCT, so the likelihood of this happening unequally between intervention and control groups is low and the potential impact on observed results is minimal.

Finally, it is important to acknowledge that a change in knowledge does not necessarily reflect a change in behavior. It was beyond the scope of this study to assess the impact of the RCT on behavior; however, an important next step would be to determine whether this app can affect behavior change in relation to burn first aid in young children with hot beverage scalds.

### Conclusions

Despite the loss to follow-up, an app-based prevention intervention for burns appears to be an effective and appealing approach for targeting mothers of young children. These results have shown that only 8 people needed to be exposed to this intervention to improve inadequate overall knowledge to adequate knowledge (ie, score of less than 4 to a score of 4) in 1 additional person. The broad reach, low cost, and scalability of this medium could potentially be feasible for other injury prevention campaigns aimed at this population, particularly given the fact that children aged 0 to 4 years are most at risk for a number of injuries that occur in the home. Additional studies are needed to determine the optimal follow-up time for this type of intervention to offset the high attrition rate noted in this intervention.

## Abbreviations

ARIA: Accessibility/Remoteness Index in Australia
IQR: interquartile range
NNT: number needed to treat
OR: odds ratio
RCT: randomized controlled trial
SEIFA: Socioeconomic Indexes for Areas
SES: socioeconomic status

## Appendix

Multimedia Appendix 1 CONSORT‐EHEALTH checklist (V 1.6.1).
